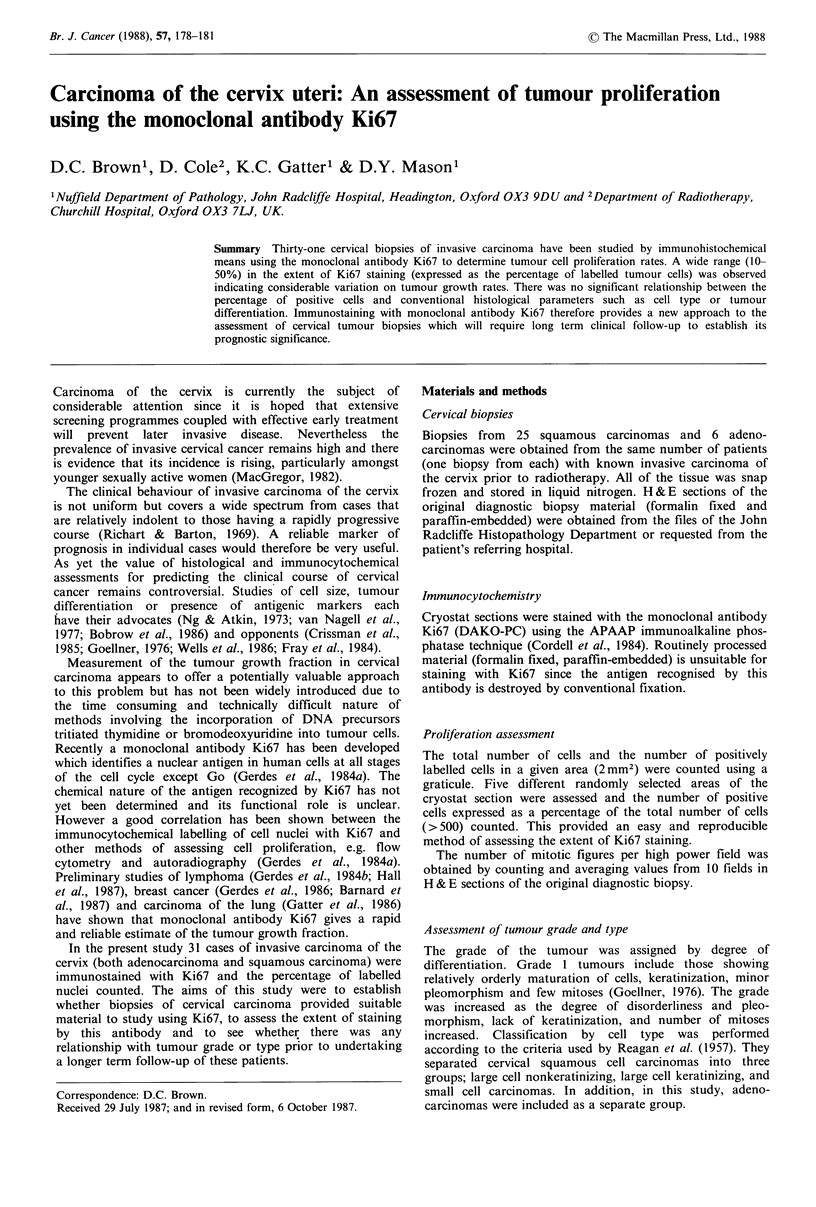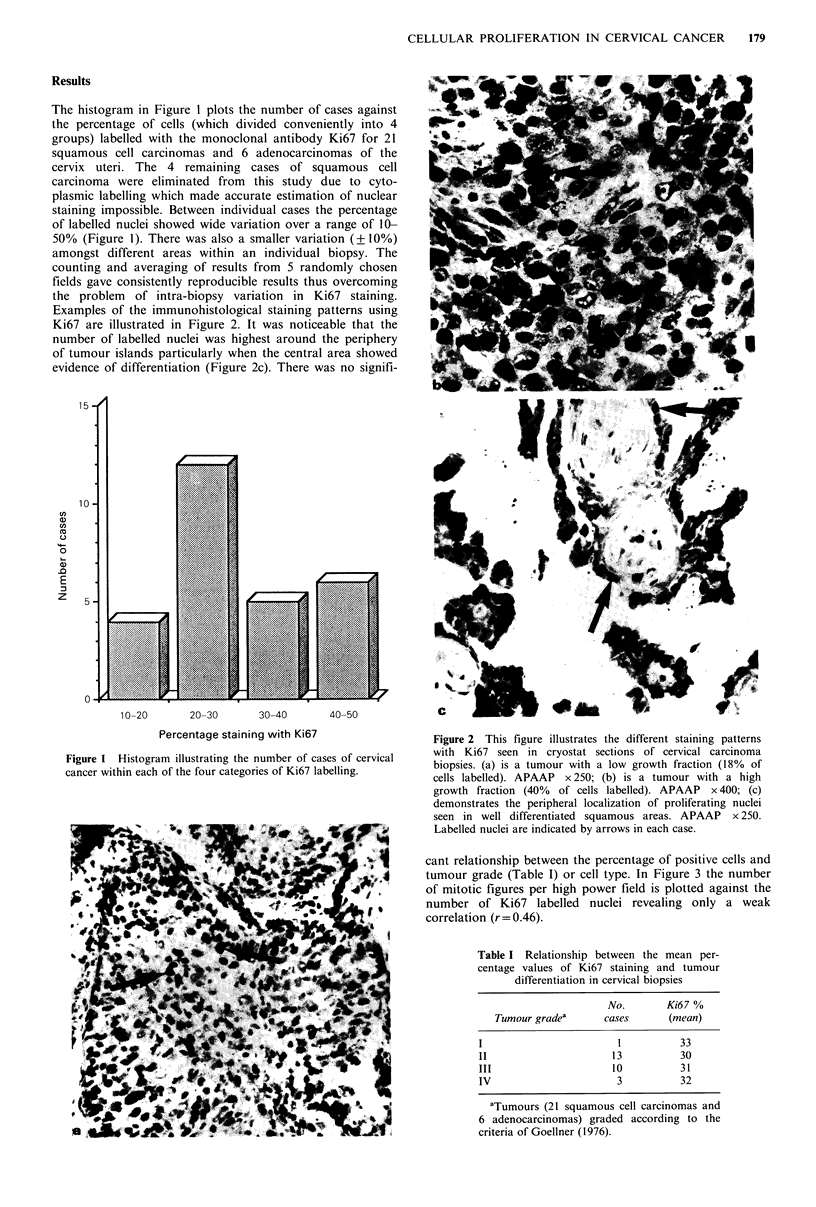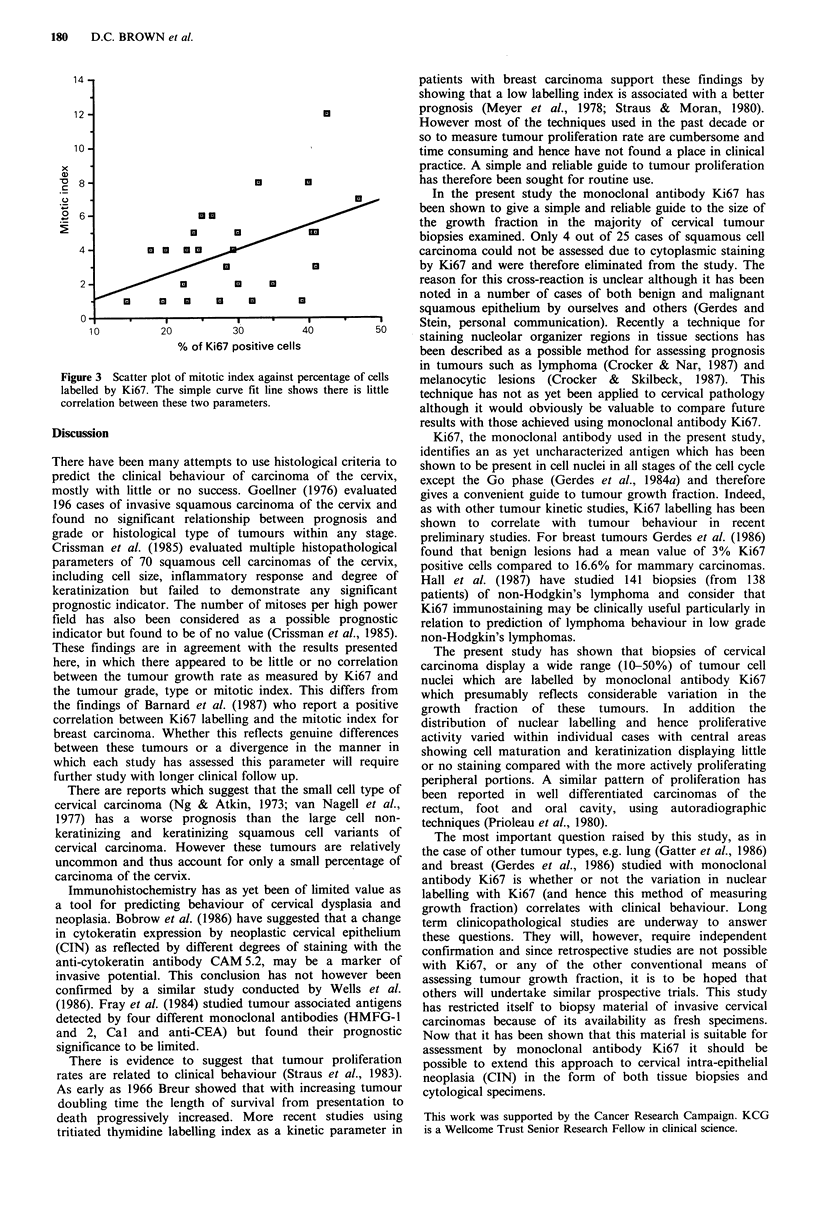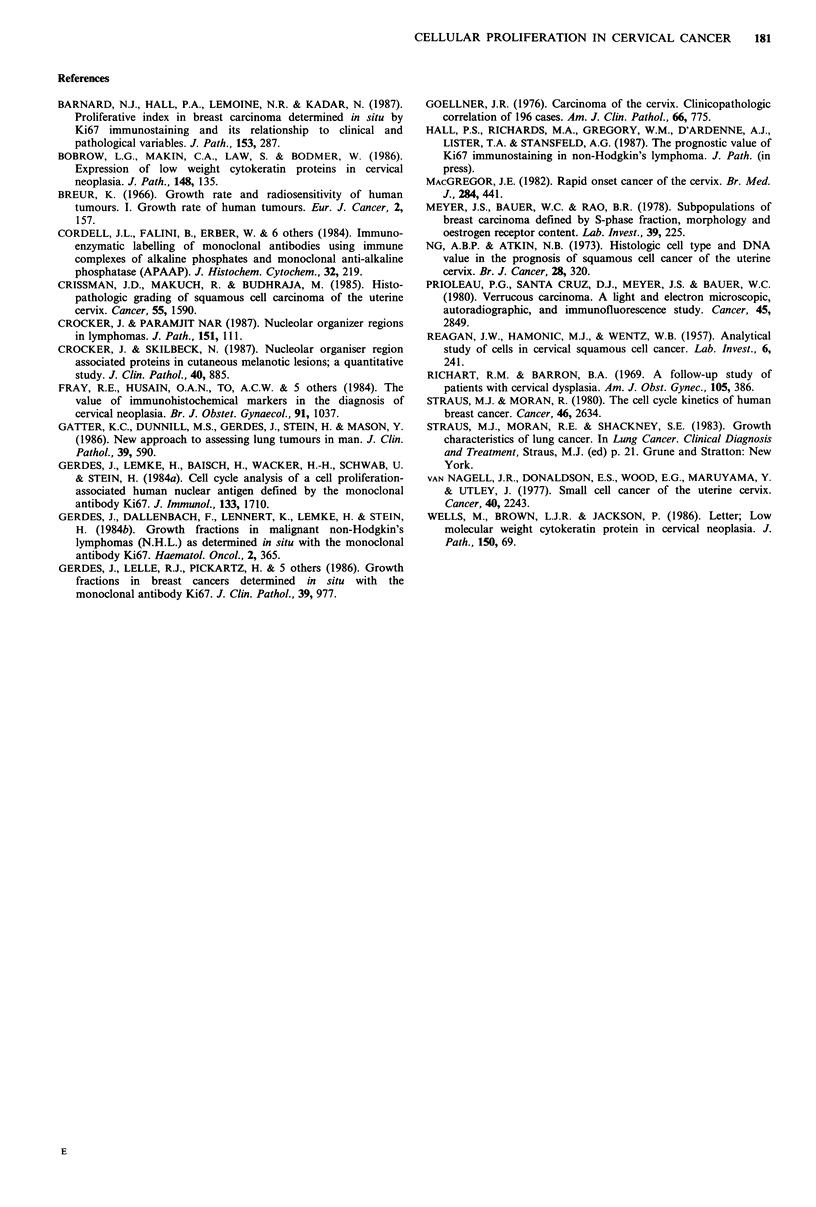# Carcinoma of the cervix uteri: an assessment of tumour proliferation using the monoclonal antibody Ki67.

**DOI:** 10.1038/bjc.1988.37

**Published:** 1988-02

**Authors:** D. C. Brown, D. Cole, K. C. Gatter, D. Y. Mason

**Affiliations:** Nuffield Department of Pathology, John Radcliffe Hospital, Headington, Oxford, UK.

## Abstract

**Images:**


*Br. J. Cancer* (1988), **57**, 178–181 © The Macmillan Press, Ltd., 1988

### Carcinoma of the cervix uteri: An assessment of tumour proliferation using the monoclonal antibody Ki67

D.C. Brown[1], D. Cole[2], K.C. Gatter[1] & D.Y. Mason[1]

[1]*Nuffield Department of Pathology, John Radcliffe Hospital, Headington, Oxford OX3 9DU and* [2]*Department of Radiotherapy, Churchill Hospital, Oxford OX3 7LJ, UK.*

**Summary** Thirty-one cervical biopsies of invasive carcinoma have been studied by immunohistochemical means using the monoclonal antibody Ki67 to determine tumour cell proliferation rates. A wide range (10–50%) in the extent of Ki67 staining (expressed as the percentage of labelled tumour cells) was observed indicating considerable variation on tumour growth rates. There was no significant relationship between the percentage of positive cells and conventional histological parameters such as cell type or tumour differentiation. Immunostaining with monoclonal antibody Ki67 therefore provides a new approach to the assessment of cervical tumour biopsies which will require long term clinical follow-up to establish its prognostic significance.

Carcinoma of the cervix is currently the subject of considerable attention since it is hoped that extensive screening programmes coupled with effective early treatment will prevent later invasive disease. Nevertheless the prevalence of invasive cervical cancer remains high and there is evidence that its incidence is rising, particularly amongst younger sexually active women (MacGregor, 1982).

The clinical behaviour of invasive carcinoma of the cervix is not uniform but covers a wide spectrum from cases that are relatively indolent to those having a rapidly progressive course (Richart & Barton, 1969). A reliable marker of prognosis in individual cases would therefore be very useful. As yet the value of histological and immunocytochemical assessments for predicting the clinical course of cervical cancer remains controversial. Studies of cell size, tumour differentiation or presence of antigenic markers each have their advocates (Ng & Atkin, 1973; van Nagell *et al.*, 1977; Bobrow *et al.*, 1986) and opponents (Crissman *et al.*, 1985; Goellner, 1976; Wells *et al.*, 1986; Fray *et al.*, 1984).

Measurement of the tumour growth fraction in cervical carcinoma appears to offer a potentially valuable approach to this problem but has not been widely introduced due to the time consuming and technically difficult nature of methods involving the incorporation of DNA precursors tritiated thymidine or bromodeoxyuridine into tumour cells. Recently a monoclonal antibody Ki67 has been developed which identifies a nuclear antigen in human cells at all stages of the cell cycle except Go (Gerdes *et al.*, 1984*a*). The chemical nature of the antigen recognized by Ki67 has not yet been determined and its functional role is unclear. However a good correlation has been shown between the immunocytochemical labelling of cell nuclei with Ki67 and other methods of assessing cell proliferation, e.g. flow cytometry and autoradiography (Gerdes *et al.*, 1984*a*). Preliminary studies of lymphoma (Gerdes *et al.*, 1984*b*; Hall *et al.*, 1987), breast cancer (Gerdes *et al.*, 1986; Barnard *et al.*, 1987) and carcinoma of the lung (Gatter *et al.*, 1986) have shown that monoclonal antibody Ki67 gives a rapid and reliable estimate of the tumour growth fraction.

In the present study 31 cases of invasive carcinoma of the cervix (both adenocarcinoma and squamous carcinoma) were immunostained with Ki67 and the percentage of labelled nuclei counted. The aims of this study were to establish whether biopsies of cervical carcinoma provided suitable material to study using Ki67, to assess the extent of staining by this antibody and to see whether there was any relationship with tumour grade or type prior to undertaking a longer term follow-up of these patients.

Correspondence: D.C. Brown.
Received 29 July 1987; and in revised form, 6 October 1987.

### Materials and methods

### Cervical biopsies

Biopsies from 25 squamous carcinomas and 6 adenocarcinomas were obtained from the same number of patients (one biopsy from each) with known invasive carcinoma of the cervix prior to radiotherapy. All of the tissue was snap frozen and stored in liquid nitrogen. H&E sections of the original diagnostic biopsy material (formalin fixed and paraffin-embedded) were obtained from the files of the John Radcliffe Histopathology Department or requested from the patient's referring hospital.

### Immunocytochemistry

Cryostat sections were stained with the monoclonal antibody Ki67 (DAKO-PC) using the APAAP immunoalkaline phosphatase technique (Cordell *et al.*, 1984). Routinely processed material (formalin fixed, paraffin-embedded) is unsuitable for staining with Ki67 since the antigen recognised by this antibody is destroyed by conventional fixation.

### Proliferation assessment

The total number of cells and the number of positively labelled cells in a given area ($2\,mm^2$) were counted using a graticule. Five different randomly selected areas of the cryostat section were assessed and the number of positive cells expressed as a percentage of the total number of cells ($>500$) counted. This provided an easy and reproducible method of assessing the extent of Ki67 staining.

The number of mitotic figures per high power field was obtained by counting and averaging values from 10 fields in H&E sections of the original diagnostic biopsy.

### Assessment of tumour grade and type

The grade of the tumour was assigned by degree of differentiation. Grade 1 tumours include those showing relatively orderly maturation of cells, keratinization, minor pleomorphism and few mitoses (Goellner, 1976). The grade was increased as the degree of disorderliness and pleomorphism, lack of keratinization, and number of mitoses increased. Classification by cell type was performed according to the criteria used by Reagan *et al.* (1957). They separated cervical squamous cell carcinomas into three groups; large cell nonkeratinizing, large cell keratinizing, and small cell carcinomas. In addition, in this study, adenocarcinomas were included as a separate group.

### Results

The histogram in Figure 1 plots the number of cases against the percentage of cells (which divided conveniently into 4 groups) labelled with the monoclonal antibody Ki67 for 21 squamous cell carcinomas and 6 adenocarcinomas of the cervix uteri. The 4 remaining cases of squamous cell carcinoma were eliminated from this study due to cytoplasmic labelling which made accurate estimation of nuclear staining impossible. Between individual cases the percentage of labelled nuclei showed wide variation over a range of 10–50% (Figure 1). There was also a smaller variation (±10%) amongst different areas within an individual biopsy. The counting and averaging of results from 5 randomly chosen fields gave consistently reproducible results thus overcoming the problem of intra-biopsy variation in Ki67 staining. Examples of the immunohistological staining patterns using Ki67 are illustrated in Figure 2. It was noticeable that the number of labelled nuclei was highest around the periphery of tumour islands particularly when the central area showed evidence of differentiation (Figure 2c). There was no significant relationship between the percentage of positive cells and tumour grade (Table I) or cell type. In Figure 3 the number of mitotic figures per high power field is plotted against the number of Ki67 labelled nuclei revealing only a weak correlation ($r=0.46$).

**Figure 1** Histogram illustrating the number of cases of cervical cancer within each of the four categories of Ki67 labelling.

**Figure 2** This figure illustrates the different staining patterns with Ki67 seen in cryostat sections of cervical carcinoma biopsies. (a) is a tumour with a low growth fraction (18% of cells labelled). APAAP ×250; (b) is a tumour with a high growth fraction (40% of cells labelled). APAAP ×400; (c) demonstrates the peripheral localization of proliferating nuclei seen in well differentiated squamous areas. APAAP ×250. Labelled nuclei are indicated by arrows in each case.

**Table I** Relationship between the mean percentage values of Ki67 staining and tumour differentiation in cervical biopsies

| *Tumour grade*[a] | *No. cases* | *Ki67 % (mean)* |
|---|---|---|
| I | 1 | 33 |
| II | 13 | 30 |
| III | 10 | 31 |
| IV | 3 | 32 |

[a]Tumours (21 squamous cell carcinomas and 6 adenocarcinomas) graded according to the criteria of Goellner (1976).

**Figure 3** Scatter plot of mitotic index against percentage of cells labelled by Ki67. The simple curve fit line shows there is little correlation between these two parameters.

### Discussion

There have been many attempts to use histological criteria to predict the clinical behaviour of carcinoma of the cervix, mostly with little or no success. Goellner (1976) evaluated 196 cases of invasive squamous carcinoma of the cervix and found no significant relationship between prognosis and grade or histological type of tumours within any stage. Crissman *et al.* (1985) evaluated multiple histopathological parameters of 70 squamous cell carcinomas of the cervix, including cell size, inflammatory response and degree of keratinization but failed to demonstrate any significant prognostic indicator. The number of mitoses per high power field has also been considered as a possible prognostic indicator but found to be of no value (Crissman *et al.*, 1985). These findings are in agreement with the results presented here, in which there appeared to be little or no correlation between the tumour growth rate as measured by Ki67 and the tumour grade, type or mitotic index. This differs from the findings of Barnard *et al.* (1987) who report a positive correlation between Ki67 labelling and the mitotic index for breast carcinoma. Whether this reflects genuine differences between these tumours or a divergence in the manner in which each study has assessed this parameter will require further study with longer clinical follow up.

There are reports which suggest that the small cell type of cervical carcinoma (Ng & Atkin, 1973; van Nagell *et al.*, 1977) has a worse prognosis than the large cell non-keratinizing and keratinizing squamous cell variants of cervical carcinoma. However these tumours are relatively uncommon and thus account for only a small percentage of carcinoma of the cervix.

Immunohistochemistry has as yet been of limited value as a tool for predicting behaviour of cervical dysplasia and neoplasia. Bobrow *et al.* (1986) have suggested that a change in cytokeratin expression by neoplastic cervical epithelium (CIN) as reflected by different degrees of staining with the anti-cytokeratin antibody CAM 5.2, may be a marker of invasive potential. This conclusion has not however been confirmed by a similar study conducted by Wells *et al.* (1986). Fray *et al.* (1984) studied tumour associated antigens detected by four different monoclonal antibodies (HMFG-1 and 2, Ca1 and anti-CEA) but found their prognostic significance to be limited.

There is evidence to suggest that tumour proliferation rates are related to clinical behaviour (Straus *et al.*, 1983). As early as 1966 Breur showed that with increasing tumour doubling time the length of survival from presentation to death progressively increased. More recent studies using tritiated thymidine labelling index as a kinetic parameter in patients with breast carcinoma support these findings by showing that a low labelling index is associated with a better prognosis (Meyer *et al.*, 1978; Straus & Moran, 1980). However most of the techniques used in the past decade or so to measure tumour proliferation rate are cumbersome and time consuming and hence have not found a place in clinical practice. A simple and reliable guide to tumour proliferation has therefore been sought for routine use.

In the present study the monoclonal antibody Ki67 has been shown to give a simple and reliable guide to the size of the growth fraction in the majority of cervical tumour biopsies examined. Only 4 out of 25 cases of squamous cell carcinoma could not be assessed due to cytoplasmic staining by Ki67 and were therefore eliminated from the study. The reason for this cross-reaction is unclear although it has been noted in a number of cases of both benign and malignant squamous epithelium by ourselves and others (Gerdes and Stein, personal communication). Recently a technique for staining nucleolar organizer regions in tissue sections has been described as a possible method for assessing prognosis in tumours such as lymphoma (Crocker & Nar, 1987) and melanocytic lesions (Crocker & Skilbeck, 1987). This technique has not as yet been applied to cervical pathology although it would obviously be valuable to compare future results with those achieved using monoclonal antibody Ki67.

Ki67, the monoclonal antibody used in the present study, identifies an as yet uncharacterized antigen which has been shown to be present in cell nuclei in all stages of the cell cycle except the Go phase (Gerdes *et al.*, 1984*a*) and therefore gives a convenient guide to tumour growth fraction. Indeed, as with other tumour kinetic studies, Ki67 labelling has been shown to correlate with tumour behaviour in recent preliminary studies. For breast tumours Gerdes *et al.* (1986) found that benign lesions had a mean value of 3% Ki67 positive cells compared to 16.6% for mammary carcinomas. Hall *et al.* (1987) have studied 141 biopsies (from 138 patients) of non-Hodgkin's lymphoma and consider that Ki67 immunostaining may be clinically useful particularly in relation to prediction of lymphoma behaviour in low grade non-Hodgkin's lymphomas.

The present study has shown that biopsies of cervical carcinoma display a wide range (10–50%) of tumour cell nuclei which are labelled by monoclonal antibody Ki67 which presumably reflects considerable variation in the growth fraction of these tumours. In addition the distribution of nuclear labelling and hence proliferative activity varied within individual cases with central areas showing cell maturation and keratinization displaying little or no staining compared with the more actively proliferating peripheral portions. A similar pattern of proliferation has been reported in well differentiated carcinomas of the rectum, foot and oral cavity, using autoradiographic techniques (Prioleau *et al.*, 1980).

The most important question raised by this study, as in the case of other tumour types, e.g. lung (Gatter *et al.*, 1986) and breast (Gerdes *et al.*, 1986) studied with monoclonal antibody Ki67 is whether or not the variation in nuclear labelling with Ki67 (and hence this method of measuring growth fraction) correlates with clinical behaviour. Long term clinicopathological studies are underway to answer these questions. They will, however, require independent confirmation and since retrospective studies are not possible with Ki67, or any of the other conventional means of assessing tumour growth fraction, it is to be hoped that others will undertake similar prospective trials. This study has restricted itself to biopsy material of invasive cervical carcinomas because of its availability as fresh specimens. Now that it has been shown that this material is suitable for assessment by monoclonal antibody Ki67 it should be possible to extend this approach to cervical intra-epithelial neoplasia (CIN) in the form of both tissue biopsies and cytological specimens.

This work was supported by the Cancer Research Campaign. KCG is a Wellcome Trust Senior Research Fellow in clinical science.

### References

BARNARD, N.J., HALL, P.A., LEMOINE, N.R. & KADAR, N. (1987). Proliferative index in breast carcinoma determined *in situ* by Ki67 immunostaining and its relationship to clinical and pathological variables. *J. Path.*, **153**, 287.

BOBROW, L.G., MAKIN, C.A., LAW, S. & BODMER, W. (1986). Expression of low weight cytokeratin proteins in cervical neoplasia. *J. Path.*, **148**, 135.

BREUR, K. (1966). Growth rate and radiosensitivity of human tumours. I. Growth rate of human tumours. *Eur. J. Cancer*, **2**, 157.

CORDELL, J.L., FALINI, B., ERBER, W. & 6 others (1984). Immunoenzymatic labelling of monoclonal antibodies using immune complexes of alkaline phosphates and monoclonal anti-alkaline phosphatase (APAAP). *J. Histochem. Cytochem.*, **32**, 219.

CRISSMAN, J.D., MAKUCH, R. & BUDHRAJA, M. (1985). Histopathologic grading of squamous cell carcinoma of the uterine cervix. *Cancer*, **55**, 1590.

CROCKER, J. & PARAMJIT NAR (1987). Nucleolar organizer regions in lymphomas. *J. Path.*, **151**, 111.

CROCKER, J. & SKILBECK, N. (1987). Nucleolar organiser region associated proteins in cutaneous melanotic lesions; a quantitative study. *J. Clin. Pathol.*, **40**, 885.

FRAY, R.E., HUSAIN, O.A.N., TO, A.C.W. & 5 others (1984). The value of immunohistochemical markers in the diagnosis of cervical neoplasia. *Br. J. Obstet. Gynaecol.*, **91**, 1037.

GATTER, K.C., DUNNILL, M.S., GERDES, J., STEIN, H. & MASON, Y. (1986). New approach to assessing lung tumours in man. *J. Clin. Pathol.*, **39**, 590.

GERDES, J., LEMKE, H., BAISCH, H., WACKER, H.-H., SCHWAB, U. & STEIN, H. (1984*a*). Cell cycle analysis of a cell proliferation-associated human nuclear antigen defined by the monoclonal antibody Ki67. *J. Immunol.*, **133**, 1710.

GERDES, J., DALLENBACH, F., LENNERT, K., LEMKE, H. & STEIN, H. (1984*b*). Growth fractions in malignant non-Hodgkin's lymphomas (N.H.L.) as determined *in situ* with the monoclonal antibody Ki67. *Haematol. Oncol.*, **2**, 365.

GERDES, J., LELLE, R.J., PICKARTZ, H. & 5 others (1986). Growth fractions in breast cancers determined *in situ* with the monoclonal antibody Ki67. *J. Clin. Pathol.*, **39**, 977.

GOELLNER, J.R. (1976). Carcinoma of the cervix. Clinicopathologic correlation of 196 cases. *Am. J. Clin. Pathol.*, **66**, 775.

HALL, P.S., RICHARDS, M.A., GREGORY, W.M., D'ARDENNE, A.J., LISTER, T.A. & STANSFELD, A.G. (1987). The prognostic value of Ki67 immunostaining in non-Hodgkin's lymphoma. *J. Path.* (in press).

MACGREGOR, J.E. (1982). Rapid onset cancer of the cervix. *Br. Med. J.*, **284**, 441.

MEYER, J.S., BAUER, W.C. & RAO, B.R. (1978). Subpopulations of breast carcinoma defined by S-phase fraction, morphology and oestrogen receptor content. *Lab. Invest.*, **39**, 225.

NG, A.B.P. & ATKIN, N.B. (1973). Histologic cell type and DNA value in the prognosis of squamous cell cancer of the uterine cervix. *Br. J. Cancer*, **28**, 320.

PRIOLEAU, P.G., SANTA CRUZ, D.J., MEYER, J.S. & BAUER, W.C. (1980). Verrucous carcinoma. A light and electron microscopic, autoradiographic, and immunofluorescence study. *Cancer*, **45**, 2849.

REAGAN, J.W., HAMONIC, M.J., & WENTZ, W.B. (1957). Analytical study of cells in cervical squamous cell cancer. *Lab. Invest.*, **6**, 241.

RICHART, R.M. & BARRON, B.A. (1969. A follow-up study of patients with cervical dysplasia. *Am. J. Obst. Gynec.*, **105**, 386.

STRAUS, M.J. & MORAN, R. (1980). The cell cycle kinetics of human breast cancer. *Cancer*, **46**, 2634.

STRAUS, M.J., MORAN, R.E. & SHACKNEY, S.E. (1983). Growth characteristics of lung cancer. In *Lung Cancer. Clinical Diagnosis and Treatment*, Straus, M.J. (ed) p. 21. Grune and Stratton: New York.

VAN NAGELL, J.R., DONALDSON, E.S., WOOD, E.G., MARUYAMA, Y. & UTLEY, J. (1977). Small cell cancer of the uterine cervix. *Cancer*, **40**, 2243.

WELLS, M., BROWN, L.J.R. & JACKSON, P. (1986). Letter; Low molecular weight cytokeratin protein in cervical neoplasia. *J. Path.*, **150**, 69.